# Diabetic retinopathy risk prediction for fundus examination using sparse learning: a cross-sectional study

**DOI:** 10.1186/1472-6947-13-106

**Published:** 2013-09-13

**Authors:** Ein Oh, Tae Keun Yoo, Eun-Cheol Park

**Affiliations:** 1 Department of Medicine, Yonsei University College of Medicine, Seoul, South Korea; 2 Department of Medical Engineering, Yonsei University College of Medicine, Seoul, South Korea; 3 Department of Preventive Medicine 6& Institute of Health Services Research, Yonsei University, Seoul, South Korea

**Keywords:** Sparse learning, LASSO, Diabetic retinopathy, Clinical decision support, Risk assessment

## Abstract

**Background:**

Blindness due to diabetic retinopathy (DR) is the major disability in diabetic patients. Although early management has shown to prevent vision loss, diabetic patients have a low rate of routine ophthalmologic examination. Hence, we developed and validated sparse learning models with the aim of identifying the risk of DR in diabetic patients.

**Methods:**

Health records from the Korea National Health and Nutrition Examination Surveys (KNHANES) V-1 were used. The prediction models for DR were constructed using data from 327 diabetic patients, and were validated internally on 163 patients in the KNHANES V-1. External validation was performed using 562 diabetic patients in the KNHANES V-2. The learning models, including ridge, elastic net, and LASSO, were compared to the traditional indicators of DR.

**Results:**

Considering the Bayesian information criterion, LASSO predicted DR most efficiently. In the internal and external validation, LASSO was significantly superior to the traditional indicators by calculating the area under the curve (AUC) of the receiver operating characteristic. LASSO showed an AUC of 0.81 and an accuracy of 73.6% in the internal validation, and an AUC of 0.82 and an accuracy of 75.2% in the external validation.

**Conclusion:**

The sparse learning model using LASSO was effective in analyzing the epidemiological underlying patterns of DR. This is the first study to develop a machine learning model to predict DR risk using health records. LASSO can be an excellent choice when both discriminative power and variable selection are important in the analysis of high-dimensional electronic health records.

## Background

A major goal of diabetic medicine is to accurately predict diabetic complications and to prevent their progression [1]. Diabetic retinopathy (DR) is the most common ocular complication of diabetes. Blindness due to retinopathy is the major disability in patients with diabetes [2]. DR is common in diabetic patients but is asymptomatic until a significant visual impairment occurs. Late diagnosis of DR results in the socio-economic burden of illness associated with diabetes [3]. Several studies have shown that early ophthalmologic examination is important in screening, diagnosing, and monitoring DR [2-4]. Early appropriate management methods such as diabetic drugs, blood pressure control, and laser photocoagulation have proven to prevent vision loss and blindness [5]. However, only a third of diabetic patients in Korea have received routine ophthalmologic examination in recent years [6]. In the U.S., approximately a half of diabetic patients do not receive any kind of examination for detecting DR although the American Diabetes Association has recommended an annual fundus examination by an ophthalmologist [7]. Therefore, clinicians face a significant challenge in identifying patients who are at a high risk of DR in a timely and appropriate manner.

DR results from diabetes-induced damage to microvascular cells. The incidence of DR is closely related to the control of serum glucose level, while other metabolic disorders also contribute to the progression of DR [2]. Recent findings suggest that an early detection of DR can be assisted by the knowledge of several biomarkers. The traditional indicators of DR included serum glucose, HbA1c (glycated hemoglobin), duration of diabetes, blood pressure, and lipid levels [8,9]. The optimal cut-off points of the traditional indicators for DR prediction have been calculated on the basis of several population-based studies [8,10]. These studies have shown that HbA1c is a more reliable predictor of DR than other traditional indicators. Other methods have been based on a combination of risk factors of DR using classical statistical methods [11,12]. However, these risk prediction methods for DR were inefficient owing to their poor prediction performance. Moreover, although these studies considered classical risk factors, they did not select important informative variables that could really contribute to DR.

Since a number of studies have shown that the pathogenesis of DR is complex and multi-factorial, understanding the whole biomarker patterns of diabetic patients will facilitate the identification of the risk of DR. However, ordinary regression shows over-fitting and instability of coefficients when a number of inter-correlated biomarkers are used [13]. Stepwise variable selection, including forward and backward stepwise selection, do not show suitability to predict disease with good discriminative ability in high-dimensional data [14]. Recently, in the field of bioinformatics, sparse learning has emerged as a tool for analyzing large-scale biomarker patterns [15,16]. Sparse learning is an area of machine learning research which can be used to find a small number of important predictors to achieve optimal prediction accuracy. Sparse learning techniques, such as least absolute shrinkage and selection operator (LASSO) and elastic net, have been widely applied to the analysis of genetic, genomic, and proteomic data [16]. When the number of variables is large or when variables are highly correlated, these techniques can offer a better regression solution than classical regression methods and other machine learning methods such as support vector machine (SVM) [15]. Due to its abilities to select important features and to detect relationships between biomarkers and diseases, sparse learning has been successfully used in medical decision support systems [16-18].

In this study, we developed and validated sparse learning models with the aim of identifying the risk of DR in diabetic patients. The objective of this study was to select diabetic patients who should receive fundus examination by an ophthalmologist in order to increase the effectiveness of screening for DR. The sparse learning techniques identified the important biomarkers to have a real effect on prediction models of DR. We compared the performance of sparse learning techniques and traditional clinical biomarkers, including HbA1c, fasting plasma glucose (FPG), and duration of diabetes.

## Methods

### Data sources

This cross-sectional study investigated prediction models for the incidence of DR. All analyses were based on the Korean National Health and Nutrition Examination Survey (KNHANES, online at http://knhanes.cdc.go.kr/). The study protocol was approved by the institutional review board at the Korea Centers for Disease Control and Prevention (IRB No: 2010-02CON-21-C, 2011-02CON-06-C). We collected health records from Korean diabetic patients based on the KNHANES V conducted in 2010 and 2011. The KNHANES V is a nationwide and population-based cross-sectional survey that was conducted by the Division of Chronic Disease Surveillance, Korea Centers for Disease Control and Prevention [19]. KNHANES consists of health records based on a health interview, a health examination, and a nutrition survey. Each participant was interviewed and asked to complete a questionnaire on his or her alcohol consumption, smoking status, diabetes mellitus, hypertension, and physical activity level. The level of physical activity was calculated using the metabolic equivalent of task values based on self-reported frequency and duration of activities during the week [20]. Height, weight, and waist circumference were measured, and the body mass index (BMI) was calculated. Measurements of HbA1c, FPG, liver enzymes, serum lipid and lipoprotein, blood urea nitrogen (BUN), and serum creatinine level were taken in local community health centers. Blood pressure (BP) was also measured by health professionals. Urinary protein, glucose, ketone, bilirubin, blood, and urobilinogen were measured by dipstick test, and urinary creatinine and sodium were measured with a chemistry analyzer. In order to assess the retinopathy status, fundus examination was done by two trained ophthalmologists according to the Early Treatment for Diabetic Retinopathy Study [21,22].

**Figure 1:**
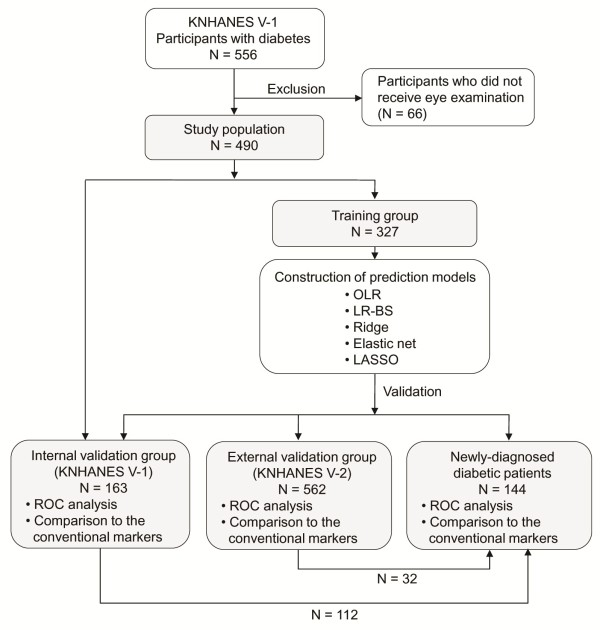
**Dataset used in the development and validation of diabetic retinopathy risk prediction.** This flowchart shows the process of training, internal validation, external validation, and validation in the newly-diagnosed diabetic patients. KNHANES, Korean National Health and Nutrition Examination Survey; LASSO, least absolute shrinkage and selection operator; LR-BS, logistic regression with backward stepwise selection; OLR, ordinary logistic regression; ROC, receiver operating characteristic.

The input variables of the prediction models were collected from demographic data, medical history, blood pressure, blood test, and urine test. The primary outcome variable was the presence of DR diagnosed by fundus examination. Data from the KNHANES V-1, conducted in 2010, was used to develop risk prediction models (Figure 1). Among 8958 participants who participated in the KNHANES V-1, 556 were diabetic patients who satisfied the diagnostic criteria of glucose level and HbA1c defined by the American Diabetes Association [23]. Diabetes was diagnosed in participants with FPG ≥ 126 mg/dL, non-fasting glucose ≥ 200 mg/dL or HbA1c ≥ 6.5%. We excluded participants who did not receive eye examination. Finally, 490 participants were included in this study, and the data from them were used as a development dataset. The development dataset was separated randomly into training and internal validation sets. The training set, comprised of two thirds (327 patients) of the entire dataset, was used to construct prediction models. The internal validation set, comprised of one third (164 patients) of the dataset, was used to assess the ability to predict DR in diabetic patients.

In order to obtain an unbiased prediction performance, the prediction models should be validated in external data. Therefore, performance of the prediction model was evaluated in independent data collected from the KNHANES V-2, conducted in 2011. Since the participants were re-selected using random sampling, the KNHANES V-2 was comprised of different participants from the KNANES V-1. Data from the KNHANES V-2 also followed the same inclusion and exclusion criteria, and 562 participants were included in the external validation dataset.

It is important to identify the patients with diabetic complications among the first-visit patients with undiagnosed diabetes, especially for clinicians [24]. Therefore, we also evaluated the discriminative ability to predict DR in newly-diagnosed diabetic patients (participants with undiagnosed diabetes). The prediction models were also validated among 144 participants (32 participants from the internal validation dataset and 112 from the external validation dataset) with undiagnosed diabetes.

### Sparse learning techniques

The form of logistic regression was used for all prediction models due to dichotomous clinical outcome. It is given as,

$$\mathrm{Logit}=\ln\left(\frac{p}{1-p}\right)=\beta_{0}+\beta_{1}X_{1}+\cdots +\beta_{i}X_{i}$$

Where ‘p’ is the probability of the disease, β_0_ is the constant and β_i_ is the coefficient of a specific predictor X_i_. This calculation of logit operator is equivalently:

$$p=\frac{1}{1+\exp^{-\mathrm{Logit}}}$$

and the likelihood L is

$$L=\prod^{n}{\;p}^{Y}{\left(1-p\right)}^{1-Y}$$

where ‘Y’ is the presence of the disease encoded as a binary categorical variable. In ordinary logistic regression (OLR), maximum likelihood estimation is used to solve for the best fitting model.

In this setting, we consider the use of penalized logistic regression methods to select predictors and to predict DR in high-dimensional clinical information data. Penalized regression methods including ridge, elastic net, and LASSO have been widely used as sparse learning tools in bioinformatics [13]. Ridge is a continuous process that shrinks coefficients and improves the prediction performance of ordinary regression [25]. However, ridge solves a fitting problem with non-zero coefficients and does not offer an easily interpretable regression model. Recently, LASSO has emerged as the most well-known sparse learning technique [26]. LASSO leads to a sparse solution of coefficients corresponding to the most important predictors, and has been known to show better performance for the prediction model selection and better identification of predictors than classical regression methods [18]. Elastic net is a generalized extension of ridge and LASSO with a mixture of ridge and LASSO penalties in likelihood function [27]. These penalized regression methods provide the stability and uniqueness of regression coefficients. In the penalized logistic regression, the general objective function for maximum likelihood estimation can be written as

$$L_{\mathrm{penalized}}=L-\mathrm{\lambda f}\left(\beta \right)$$

where the penalty component f(β) is a function of the regression coefficients and λ is the sparseness control parameter. In this study, we used the Glmnet software [27,28]. In this software, the objective function of the penalized logistic regression is

$$L_{\mathrm{penalized}}=L-\lambda \left(\frac{1-\alpha }{2}|\left|\beta \right|{|}_{2}^{2}+\alpha |\left|\beta \right|{|}_{1}\right)$$

Where $|\left|\beta \right|{|}_{2}^{2}=\sum \beta_{i}^{2}$ and $|\left|\beta \right|{|}_{1}=\sum \left|\beta_{i}\right|$ are the penalty functions of ridge and LASSO, respectively. The mixing parameter α determines the strength of the penalty components of ridge and LASSO. When α = 0, this problem is equivalent to ridge regression. If 0 < α < 1, this formulation is used to solve the regression problem inelastic net. We implemented elastic net with α = 0.4 according to a previous study which included the penalized logistic regression [28]. When α = 1, this problem is equivalent to LASSO. We obtained the optimized solutions of each penalized logistic regression using Glmnet.

In all penalized regression methods, it is necessary to determine the sparseness control parameter λ. In the training dataset, we designed the 5-fold cross validation not only to assess performance, but also to optimize λ. The area under the curve (AUC) of the receiver operating characteristic (ROC) is known as a strong predictor of performance, especially with regard to imbalanced problems. Due to the imbalanced data in this study, prediction accuracy might not be a good criterion for assessing performance since the minor class has less influence on accuracy than the major class. Therefore, we investigated the AUC during the 5-fold cross validation as λ increased. The λ that indicated the highest AUC was chosen for the final training condition.

The relative importance of predictors was estimated by standardized regression coefficients of sparse learning. Standardized regression coefficients were calculated using standardized input variables, and facilitated the interpretation and comparison of the relative importance of predictors [29]. A predictor is more important to predict DR if it has a larger standardized regression coefficients.

In order to compare the performance of the sparse learning techniques, classical regression method including OLR and logistic regression with backward stepwise selection (LR-BS) were also constructed using the same training dataset.

### Model selection and validation

We constructed five prediction models including OLR, LR-BS (with a significance of 0.1 to remove the non-significant variables), ridge, elastic net, and LASSO. After training process with the whole training dataset, in order to select the best prediction model, we evaluated diagnostic abilities based on the Bayesian information criterion (BIC) in the internal validation dataset. BIC is widely used in model selection and an effective indicator to compare the prediction performance when different numbers of covariates or predictors are included in the prediction models [30]. BIC penalizes the number of variables to avoid an unstable or over-fitting model [31]. In our study, BIC can be written as:

$$\mathrm{BIC}=-2\log\left(L\right)+k\;\log\left(n\right)$$

where ‘L’ is likelihood of the prediction model in internal validation dataset, ‘k’ is the number of predictors, and ‘n’ is the number of samples of internal validation dataset. The best model is the one that gives the lowest BIC value, which means the largest marginal likelihood of data. We finally tested the selected prediction models on the internal and external validation groups using ROC analysis.

Three different clinical scenarios were developed in this study. Scenario 1 was based on the clinical variables, except the laboratory measurements. The variables obtained from the anthropometric measurements, medical history, and blood pressures were entered in the scenario. In this scenario, invasive procedure for blood sampling is not required. Therefore, the prediction models were designed for use in simple setting to predict an individual's risk using clinical variables that can be self-assessed or easily identified by the public health center. Scenario 2 was developed by adding the result of blood test to scenario 1. Since diabetes mellitus is generally diagnosed by plasma glucose level or HbA1c from blood test, this scenario was done to evaluate the effect of general clinical information obtained from clinicians’ practice. Scenario 3 was developed by adding the result of urine test to scenario 2. When the patient visits to the clinic for diabetes, urine test is routinely performed to detect diabetic nephropathy. We evaluated the additional effect of clinical information from the urine test in this scenario.

The final prediction models were validated in two populations: the KNHANES V-1 (internal validation group) and the KNHANES V-2 (external validation group). The AUC, accuracy, sensitivity, and specificity of the sparse learning models and the traditional clinical biomarkers were calculated in ROC analysis. We generated the ROC curves and selected cut-off points that maximized Youden's index [32]. Participants above the cut-off points were classified as being at high risk in each prediction model. We used SPSS 18.0 (SPSS Inc., Chicago, IL) for statistical analysis and MedCalc 12.3 (MedCalc, Mariakerke, Belgium) for ROC analysis.

## Results

The background characteristics of the development dataset (KNHANES V-1) are presented in Table 1. Eighty-four (17.1%) of 490 diabetic patients had DR. By comparison with the patients in the control group, diabetic patients in the development dataset were of significantly higher duration of diabetes, HbA1c, and FPG, and were of significantly lower BMI, diastolic BP, hemoglobin, and urine sodium level. Diabetic patients were more likely to have proteinuria, glycosuria, and diabetic histories including diagnosed diabetes, insulin therapy, anti-diabetic drug, and nondrug anti-diabetic therapy.

**Table 1:** Characteristics of the patients with diabetes mellitus in the development dataset (KNHANES V-1)

		**Total diabetic patients (N = 490)**	**Without diabetic retinopathy (N = 406)**	**With diabetic retinopathy (N = 84)**	***p*-value**
**Demographics**					
	Sex (male : female)	253 : 237	211 : 195	42 : 42	0.742^†^
	Age (years)	60.8 ± 11.7	60.7 ± 11.7	61.4 ± 11.6	0.463^¶^
	Current smoke	107 (21.8)	84 (20.7)	23 (27.4)	0.177^†^
	Alcohol (>1 serving/week)	196 (40.0)	166 (40.9)	30 (35.7)	0.378^†^
	Physical activity (MET h/week)	14.6 ± 13.0	14.2 ± 12.01	16.4 ± 16.6	0.673^¶^
	Waist circumference (cm)	87.1 ± 9.7	87.4 ± 9.9	85.5 ± 8.3	0.112^‡^
	BMI (kg/m^2^)	25.0 ± 3.3	25.2 ± 3.3	23.8 ± 3.3	0.001^‡^
**Medical history**					
	Duration of diabetes (years)	6.2 ± 7.4	5.2 ± 6.7	10.6 ± 8.7	<0.001^¶^
	Diagnosed diabetes	331 (67.6)	252 (62.1)	79 (94.1)	<0.001^†^
	Insulin therapy	30 (6.1)	15 (3.7)	15 (17.9)	<0.001^†^
	Anti-diabetic drug	297 (60.6)	233 (57.4)	64 (76.2)	<0.001^†^
	Nondrug anti-diabetic therapy	331 (67.6)	252 (62.1)	79 (94.1)	<0.001^†^
	Diagnosed hypertension	267 (54.5)	228 (56.2)	39 (46.4)	0.103^†^
	Drug for hypertension	254 (51.8)	216 (53.2)	38 (45.2)	0.184^†^
	Diagnosed hyperlipidemia	145 (29.6)	118 (29.1)	27 (18.6)	0.574^†^
	Drug for hyperlipidemia	101 (20.6)	84 (20.7)	17 (20.2)	0.926^†^
**Blood pressure**					
	Systolic BP (mmHg)	126.2 ± 16.4	126.5 ± 16.1	124.9 ± 17.8	0.413^‡^
	Diastolic BP (mmHg)	75.3 ± 9.8	76.0 ± 9.7	72.1 ± 9.9	0.001^¶^
**Blood test**					
	HbA1c (%)	7.3 ± 1.5	7.1 ± 1.5	7.9 ± 1.5	<0.001^¶^
	FPG (mg/dL)	139.3 ± 42.5	136.8 ± 40.5	151.2 ± 49.5	0.008^¶^
	AST (IU/L)	25.4 ± 12.8	25.3 ± 12.5	25.8 ± 14.4	0.408^¶^
	ALT (IU/L)	26.3 ± 16.4	26.3 ± 16.1	25.9 ± 17.6	0.297^¶^
	Hemoglobin (g/dL)	14.1 ± 1.5	14.2 ± 1.5	13.6 ± 1.7	0.003^¶^
	Cholesterol (mg/dL)	186.3 ± 40.8	185.9 ± 39.3	187.9 ± 47.5	0.836^¶^
	HDL (mg/dL)	47.8 ± 12.1	48.0 ± 12.1	46.9 ± 12.1	0.370^¶^
	LDL (mg/dL)	109.6 ± 34.3	109.6 ± 33.8	109.7 ± 36.8	0.827^¶^
	TG (mg/dL)	180.5 ± 172.7	172.9 ± 124.8	217.4 ± 313.0	0.218^¶^
	BUN (mg/dL)	15.8 ± 5.0	15.7 ± 4.9	16.2 ± 5.3	0.543^¶^
	Serum creatinine (mg/dL)	0.87 ± 0.27	0.86 ± 0.26	0.89 ± 0.30	0.709^¶^
**Urine test**					
	Protein* (+)	64 (13.1)	44 (10.8)	20 (23.8)	0.001^†^
	Glucose* (+)	123 (25.1)	90 (22.2)	33 (39.3)	0.001^†^
	Ketone* (+)	59 (12.0)	48 (11.8)	11 (13.1)	0.744^†^
	Bilirubin* (+)	58 (11.8)	45 (11.1)	13 (15.5)	0.257^†^
	Blood* (+)	169 (34.5)	133 (32.8)	36 (42.9)	0.076^†^
	Urobilinogen* (+)	6 (1.2)	4 (1.0)	2 (0.4)	0.274^§^
	Urine creatinine (mg/L)	123.3 ± 69.9	125.9 ± 70.8	110.8 ± 64.2	0.051^¶^
	Urine sodium (mmol/day)	124.5 ± 47.2	126.6 ± 48.1	114.3 ± 41.5	0.037^¶^

*by Dipstick (0, negative; 1, positive).

*p*-value were obtained by ^†^Chi-squared test, ^§^Fisher’s exact test, ^¶^Mann–Whitney test, and ^‡^Student t-test.

Table values are given as mean ± standard deviation or number (%) unless otherwise indicated.

*ALT* Alanine aminotransferase *AST* Aspartate aminotransferase, *BMI* Body mass index, *BP* Blood pressure, *BUN* Blood urea nitrogen, *FPG* Fasting plasma glucose, *HbA1c* Glycated hemoglobin, *HDL* High-density lipoprotein, *LDL* Low-density lipoprotein, *TG* Triglyceride.

**Figure 2:**
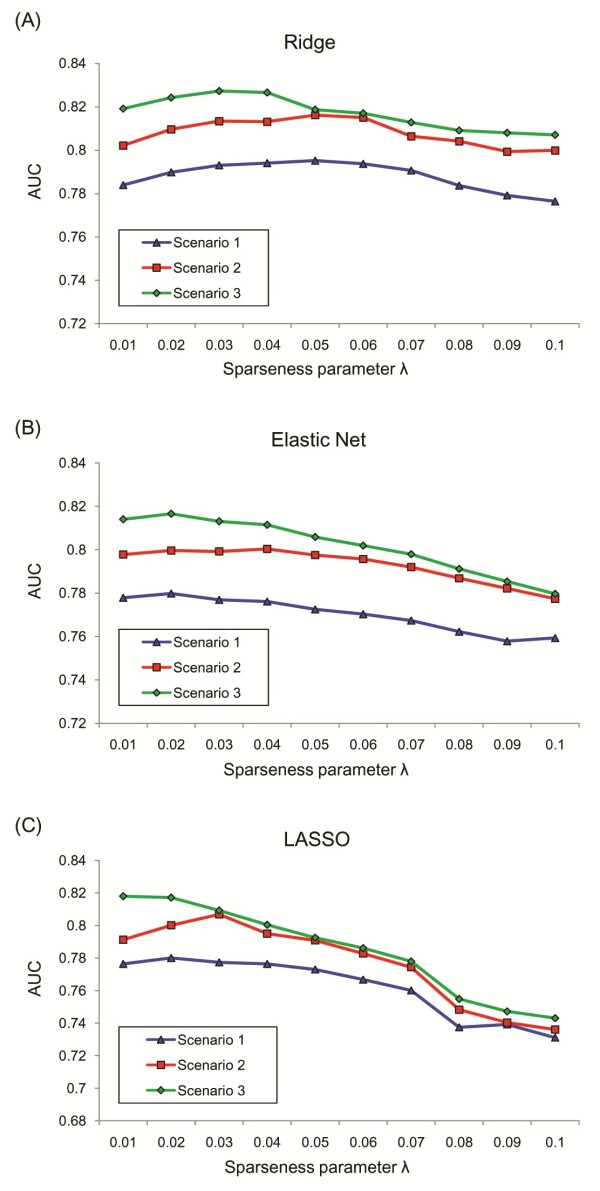
**Performance (AUC) of the penalized logistic regression models using the 5-fold cross validation.** The penalized logistic regression models included ridge **(A)**, elastic net **(B)**, and LASSO **(C)**. In order to optimize λ, we investigated the AUC during the 5-fold cross validation as λ increased. The λ that indicated the highest AUC was chosen for the final training condition.

Figure 2 shows the AUC of the penalized logistic regression models using the 5-fold cross validation as λ is varied. We found that the optimal values of λ were different in the different clinical scenarios. When the optimal values of λ were applied for training the penalized logistic regression including ridge, elastic net, and LASSO, the resulting coefficients models that we obtained are given in Additional file 1. The coefficients of OLR and LR-BS were also calculated. While OLR and ridge utilized all variables, LR-BS showed the smallest number of predictors among the five prediction methods–10, 12, and 16 predictors in scenarios 1, 2, and 3, respectively. The most popular sparse learning technique, that is, LASSO, selected 12, 14, and 19 variables as important predictors in scenarios 1, 2, and 3, respectively. Table 2 shows the standardized coefficients of the final LASSO prediction model. In scenario 1, duration of diabetes was the most important predictor. In scenarios 2 and 3, which included the results of blood test, FPG was the most important predictor.

**Table 2:** Diabetic retinopathy risk predictors identified by LASSO

		**Scenario 1 (DE + MH + BP)**		**Scenario 2 (DE + MH + BP + BT)**		**Scenario 3 (DE + MH + BP + BT + UT)**		**Reference No.**
		β*	**Std *β***^**†**^	β*	**Std *β***^**†**^	β*	**Std *β***^**†**^	
**Demographics**								
	Sex (female)	0.199	0.099					[50]
	Age (years)	−0.022	−0.260	−0.007	−0.077	−0.012	−0.146	[42,43]
	Current smoke	0.216	0.088	0.532	0.218	0.469	0.192	[2,41]
	Alcohol (>1 serving/week)	−0.039	−0.019	−0.159	−0.077	−0.137	−0.066	[48]
	Physical activity (MET h/week)	0.005	0.058					[41]
	BMI (kg/m^2^)	−0.058	−0.197	−0.059	−0.202	−0.082	−0.281	[44]
**Medical history**								
	Duration of diabetes (years)	0.054	0.376	0.027	0.186	0.027	0.187	[2,50]
	Diagnosed diabetes	0.242	0.100	0.592	0.244	0.427	0.176	[2]
	Insulin therapy	1.012	0.237	1.117	0.261	0.956	0.224	[45]
	Diagnosed hypertension	−0.228	−0.227					[2]
	Drug for hyperlipidemia	−0.036	−0.014			−0.028	−0.011	[2,41]
**Blood pressure**								
	Diastolic BP (mmHg)	−0.007	−0.067	−0.007	−0.066	−0.004	−0.041	[9]
**Blood test**								
	HbA1c (%)			0.103	0.155	0.054	0.081	[2,8]
	FPG (mg/dL)			0.009	0.402	0.008	0.339	[2]
	Hemoglobin (g/dL)			−0.230	−0.036	−0.256	−0.040	[41]
	TG (mg/dL)			0.002	0.298	0.002	0.322	[47,50]
	HDL (mg/dL)			−0.003	−0.030			[47]
	BUN (mg/dL)			0.037	0.177	0.037	0.181	[46]
**Urine test**								
	Protein (+)					0.141	0.148	[24,43]
	Glucose (+)					0.442	0.191	[24]
	Ketone (+)					−0.111	−0.036	
	Bilirubin (+)					0.118	0.041	
	Blood (+)					0.096	0.046	

*Regression coefficient of logit operator.

^†^Standardized regression coefficient of logit operator.

*BMI* Body mass index, *BP* Blood pressure, *BT* Blood test, *BUN* Blood urea nitrogen, *DE* Demographics, *FPG* Fasting plasma glucose, *HbA1c* Glycated hemoglobin, *HDL* High-density lipoprotein, *MH* Medical history, *TG* Triglyceride, *UT* Urine test.

**Figure 3:**
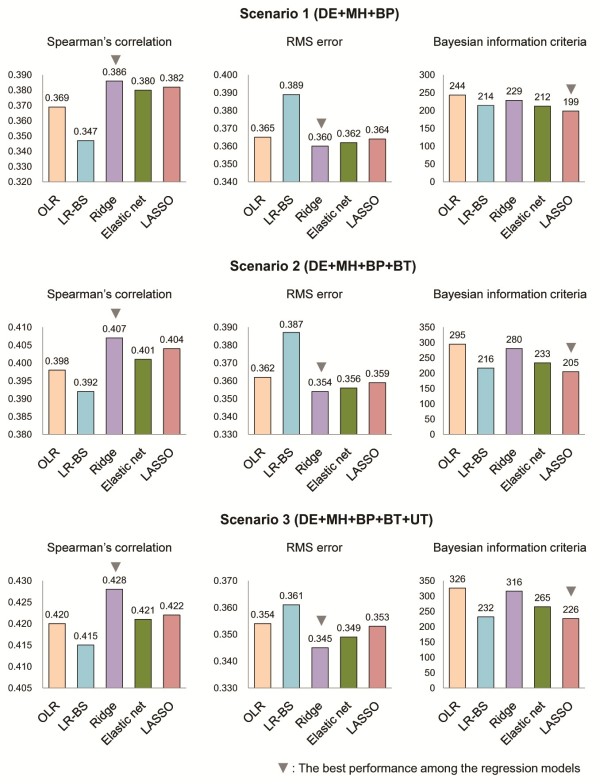
**Performance comparison of the prediction models in the internal validation group.** BP, blood pressure; BT, blood test; DE, demographics; LASSO, least absolute shrinkage and selection operator; LR-BS, logistic regression with backward stepwise selection; MH, medical history; OLR, ordinary logistic regression; RMS, root mean square; UT, urine test.

To assess the ability of the models for predicting DR, we applied our methods to a testing set composed of the independent data from the training set. Figure 3 describes the performance comparison of the prediction models in the internal validation group. We found that the penalized logistic regression methods showed better discriminative ability than OLR and LR-BS. Ridge had the smallest root mean square error and the highest Spearman's correlation value. When we investigated BIC to consider the effectiveness of the prediction models, LASSO showed a lower value of BIC than other methods. In other words, the LASSO model predicted DR most efficiently. Application of the BIC criteria to DR prediction resulted in a final LASSO model of fewer independent predictors with only a small loss in discrimination than ridge and elastic net.

**Figure 4:**
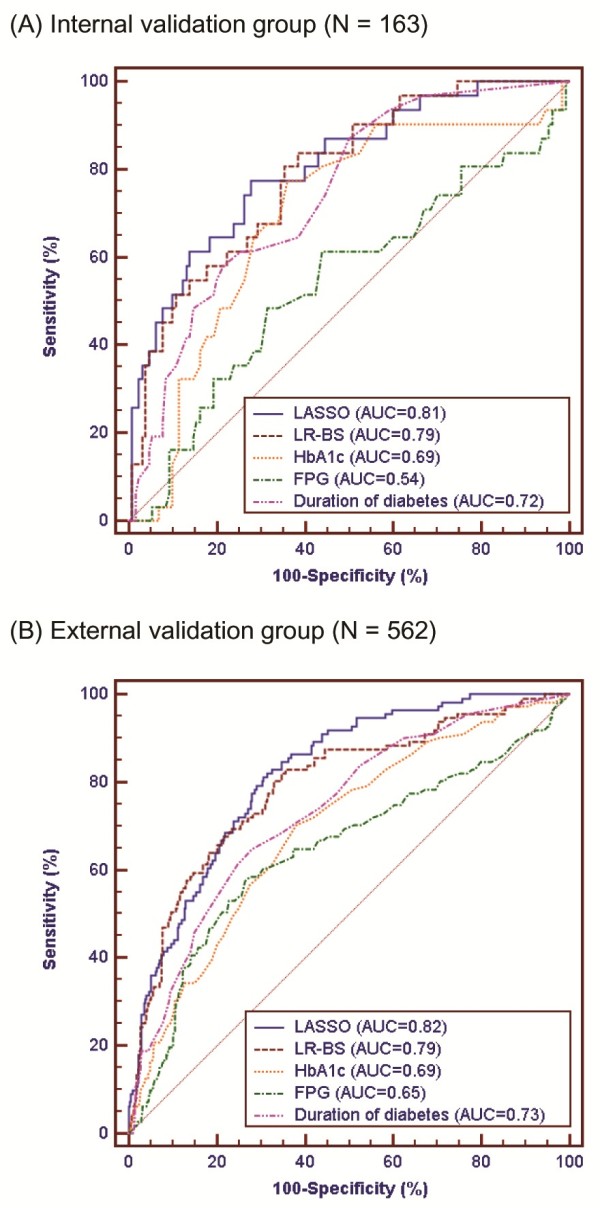
**ROC curves for diabetic retinopathy prediction.** The prediction models were tested in the internal **(A)** and external **(B)** validation groups. The LASSO and LR-BS models were trained in scenario 3. FPG, fasting plasma glucose; HbA1c, glycated hemoglobin; LASSO, least absolute shrinkage and selection operator; LR- BS, logistic regression with backward stepwise selection.

Additionally, we analyzed ROC curves of our methods in the internal and external validation datasets. Because LASSO and LR-BS had the lowest BIC among the penalized regression methods and classical methods, respectively, we compared their ROC curves. The traditional clinical biomarkers, including HbA1c, FPG, and duration of diabetes, were also included for comparison with LASSO and LR-BS developed in scenario 3. As a result, the LASSO model was the best discriminator between the control diabetic patients and the patients with DR (Figure 4). In the internal validation dataset, the LASSO model yielded an AUC of 0.81, accuracy of 73.6%, sensitivity of 77.4%, and specificity of 72.7%. Consistent results were observed in the external validation dataset. The LASSO predicted DR with an AUC of 0.82, accuracy of 75.2%, sensitivity of 72.1%, and specificity of 76.0% in the external validation. Considering AUC as a performance metric, the LASSO was significantly superior to the HbA1c, FPG, and duration of diabetes in both the internal and external validation (Table 3). The ROC analysis results of the LASSO models in scenarios 1 and 2 are shown in Additional file 2.

**Table 3:** Diagnostic performance of prediction models in the internal and external validation groups

		**AUC (95% CI)**	**Accuracy (%) (95% CI)**	**Sensitivity (%) (95% CI)**	**Specificity (%) (95% CI)**	**PPV (%)**	**NPV (%)**
(A) Internal validation group (N = 163)							
	LASSO^†^	0.81 (0.74-0.86)	73.6 (66.0-80.1)	77.4 (70.1-83.5)	72.7 (65.1-79.3)	40.0	93.2
	LR-BS^†^	0.79 (0.72-0.85)	64.4* (56.5-71.7)	83.9 (77.1-89.1)	59.8 (51.9-67.4)	32.9	94.0
	HbA1c	0.69* (0.62-0.76)	66.3* (58.4-73.4)	77.4 (70.1-83.5)	63.6 (55.7-70.9)	33.3	92.3
	FPG	0.54* (0.46-0.62)	57.7* (49.7-65.3)	61.3 (53.3-68.7)	56.8 (48.8-64.5)	25.0	86.2
	Duration of diabetes	0.72* (0.66-0.79)	57.1* (49.1-64.7)	87.1 (80.7-91.7)	50.0 (42.1-57.9)	29.0	94.3
(B) External validation group (N = 562)							
	LASSO^†^	0.82 (0.78-0.85)	75.2 (71.3-78.7)	72.1 (68.0-75.8)	76.0 (72.1-79.5)	43.7	91.3
	LR-BS^†^	0.79 (0.75-0.83)	68.7* (64.6-72.6)	82.0 (78.4-85.1)	65.3 (61.1-69.3)	37.9	93.3
	HbA1c	0.69* (0.65-0.73)	63.7* (59.5-67.7)	70.3 (66.2-74.1)	62.0 (57.7-66.1)	32.4	89.0
	FPG	0.65* (0.60-0.69)	68.3* (64.1-72.0)	57.7 (53.4-61.8)	73.4 (69.4-77.1)	36.0	87.0
	Duration of diabetes	0.73* (0.69-0.77)	69.6* (66.5-74.3)	64.9 (60.7-68.9)	72.0 (68.0-75.7)	37.5	88.8

*AUC or accuracy is significantly different from the LASSO at the level of *p* < 0.05.

^†^The LASSO and LR-BS models were trained in scenario 3.

*AUC* Area under the receiver operating characteristic curve, *CI* Confidence interval, *FPG* Fasting plasma glucose, *HbA1c* Glycated hemoglobin, *LASSO* Least absolute shrinkage and selection operator, *LR-BS* Logistic regression with backward stepwise selection, *NPV* Negative predictive value, *PPV* Positive predictive value.

The LASSO and LR-BS prediction models were also validated among 144 participants who had undiagnosed diabetes (Table 4). Although the newly-diagnosed diabetic patients had the same value of zero-duration of diabetes, the prediction models showed a similar performance to the results in Table 3. The LASSO predicted DR with an AUC of 0.90, accuracy of 89.2%, sensitivity of 75.0%, and specificity of 89.6% in the newly-diagnosed diabetic patients. Based on these findings, if we assume that 1,000 first-visit participants with undiagnosed diabetes will be examined by the LASSO model, then, 196 cases of diabetic patients would be accurately identified as having retinopathy or non-retinopathy when compared to HbA1c that is the most reliable traditional marker.

**Table 4:** Diagnostic performance of prediction models in the newly-diagnosed diabetic patients in the total validation group

	**AUC (95% CI)**	**Accuracy (%) (95% CI)**	**Sensitivity (%) (95% CI)**	**Specificity (%) (95% CI)**	**PPV (%)**	**NPV (%)**
LASSO*	0.90 (0.84-0.95)	89.2 (82.8-93.6)	75.0 (67.1-81.6)	89.6 (83.2-93.9)	16.7	99.2
LR-BS*	0.85 (0.79-0.91)	72.3 (64.2-79.2)	100.0 (96.8-100.0)	71.5 (63.4-78.5)	8.9	100.0
HbA1c	0.64 (0.55-0.72)	69.6 (61.4-76.8)	62.5 (54.2-70.8)	70.1 (62.0-77.3)	4.4	98.1
FPG	0.73 (0.65-0.80)	65.5 (57.2-73.1)	75.0 (67.1-81.6)	65.3 (57.0-72.8)	5.7	98.9

*The LASSO and LR-BS models were trained in scenario 3.

*AUC* Area under the receiver operating characteristic curve, *CI* Confidence interval, *FPG* Fasting plasma glucose, *HbA1c* Glycated hemoglobin, *LASSO* Least absolute shrinkage and selection operator, *LR-BS* Logistic regression with backward stepwise selection, *NPV* Negative predictive value, *PPV* Positive predictive value.

To show the effectiveness of the proposed method in predicting DR, we also implemented several commonly used algorithms from the literature [33,34]. Five algorithms, including SVM, artificial neural network (ANN), Random forest, Naïve Bayes classifier, and k-Nearest Neighbors, were tested on the same validation dataset (Table 5). Among these algorithms, the best prediction was provided by SVM in both the internal and external validation dataset. LASSO showed a similar but slightly lower AUC value than SVM in the internal validation dataset. In the external validation dataset, LASSO outperformed the other algorithms. We found no statistically significant difference in the AUC between LASSO and SVM (*p* = 0.304 and 0.684 in the internal and external validation dataset, respectively).

**Table 5:** Diagnostic performance of the commonly used algorithms in the literatures

Methods*		**AUC (95% CI)**	**Accuracy (%) (95% CI)**	**Sensitivity (%) (95% CI)**	**Specificity (%) (95% CI)**	**PPV (%)**	**NPV (%)**
(A) Internal validation group (N = 163)							
	SVM (RBF kernel)	0.83 (0.76-0.88)	74.8 (67.3-81.2)	71.0 (63.3-77.7)	75.8 (68.3-82.0)	40.7	91.7
	ANN	0.79 (0.72-0.85)	71.2 (63.5-77.9)	80.6 (73.6-86.3)	68.9 (61.2-75.9)	37.9	93.8
	Random Forest	0.80 (0.73-0.85)	72.4 (64.8-79.0)	87.1 (80.7-91.7)	68.9 (61.2-75.9)	39.7	95.8
	Naïve Bayes	0.76 (0.69-0.82)	74.2 (66.7-80.7)	74.2 (66.6-80.6)	74.2 (66.7-80.7)	40.4	92.5
	*k*-Nearest Neighbors	0.52 (0.45-0.59)	71.2 (63.5-77.9)	16.1 (11.0-22.8)	84.1 (77.4-89.3)	19.2	81.0
(B) External validation group (N = 562)							
	SVM	0.81 (0.78-0.84)	74.1 (70.1-77.7)	75.7 (71.8-79.2)	73.7 (69.7-77.3)	42.6	92.1
	ANN	0.79 (0.76-0.83)	71.9 (67.8-75.6)	81.1 (77.5-84.3)	69.5 (65.4-73.3)	40.7	93.4
	Random Forest	0.76 (0.72-0.79)	71.1 (67.1-74.9)	69.4 (65.3-73.2)	71.6 (67.5-75.3)	38.7	90.0
	Naïve Bayes	0.73 (0.69-0.77)	70.6 (66.5-74.3)	69.4 (65.3-73.2)	70.9 (66.8-74.6)	38.1	89.9
	*k*-Nearest Neighbors	0.52 (0.48-0.57)	73.7 (69.7-77.3)	16.2 (13.3-19.6)	88.6 (85.5-91.1)	26.9	80.3

*The models were trained and validated in scenario 3 without feature selection. The optimal conditions of each method were obtained in the 5-fold cross validation.

*ANN* Artificial neural network, *AUC* Area under the receiver operating characteristic curve, *CI* Confidence interval, *NPV* Negative predictive value, *PPV* Positive predictive value, *RBF* Radial basis function, *SVM* Support vector machine.

## Discussion

In this study, we introduced a bioinformatics-inspired method using sparse learning techniques in order to predict DR risk among diabetic patients. Our proposed LASSO model was designed for use in the self-assessment setting (scenario 1) and in the clinical setting with better prediction of DR (scenarios 2 and 3). Consistent results were observed when we applied the prediction model to the newly-diagnosed diabetic patients. We expect that the clinical information recorded in the electronic health records can be easily used by our proposed method for identifying diabetic populations who are at a high risk of DR. To our knowledge, this is the first study to develop a sparse learning model for DR risk prediction using population-based health records. No prior report has investigated the ability of machine learning to predict DR risk in a clinical manner.

The LASSO, the most well-known sparse learning technique, predicted DR most efficiently. ROC analysis supported that the LASSO model had a statistically significant improvement in predicting DR. This finding is consistent with previous studies on the comparison of sparse learning and conventional methods in various complex discriminating problems for predicting disease with genetic data [17,35]. The strengths of our proposed sparse learning method are feature selection, good prediction performance, and easily interpretable results. Several studies have earlier pointed out that SVM and ANN can be considered an incomprehensible black-box due to its complexity [36,37]. Whereas, the LASSO model is composed of easily interpretable regression coefficients that can provide an insight into risk factors of DR. In our study, LASSO offered a prediction model with important predictor selection as well as a similar performance to SVM, which has shown to perform well in multiple research areas lately [15]. Therefore, LASSO can be an excellent choice when both discriminative power and variable selection are important in a high-dimensional clinical problem [38].

The need for fundus examination at the time of diagnosis of diabetes has been confirmed [2,39]. However, several reports have revealed that many patients neglect ophthalmologic examination due to asymptomatic eye status in the early stage and poor access to ophthalmologic care, and that the rate of referrals from primary care physicians to ophthalmologists is low [6,40]. We expect that our method will be especially useful in the population with poor access to ophthalmologic care. Most experts predicted DR risk using level of HbA1c or FPG [8]. However, our study has shown the poor performance of HbA1c and FPG in predicting DR. Our proposed sparse learning method has shown significantly better performance than HbA1c and FPG. If the LASSO prediction model retains good performance after validation in a larger population, it will be possible to use this technique to determine candidates for evaluation with fundus examination and also to prevent visual impairment due to progression of DR.

In this study, when all clinical data were available, in the LASSO model, the presence of DR was associated with the 19 predictors. LASSO algorithm identified FPG, triglyceride (TG), low BMI, and insulin therapy as strong predictors (absolute standardized regression coefficient > 0.2). In general, smoking, anemia (low level of hemoglobin), high level of HbA1c, FPG, and TG were shown as the common modifiable risk factors of DR in previous studies [8,41]. The young age of the diabetic patients was also an important predictor. Several studies have shown that diagnosis of diabetes at a young age is closely related to long duration of diabetes and DR [42,43]. Long duration of diabetes may affect the severity of diabetes that is associated with weight loss (low BMI) and history of insulin therapy [44,45]. Since diabetic nephropathy is also caused by microvascular damage, the biomarkers of kidney dysfunction, including elevated BUN and urine dipstick test positive, are closely related to DR [24,46,47]. We found that low diastolic blood pressure was a better predictor of DR in contrast with several previous studies [2]. There is a theoretical basis for assuming that pulse pressure (systolic pressure-diastolic pressure) has stronger effect on microvascular cell damage than simple hypertension [9]. Therefore, it is possible that diabetic complications could be affected by low diastolic pressure. Alcohol consumption was also an unexpected factor. Recent studies have proposed that moderate alcohol consumption may prevent cardiovascular complications in diabetic patients [48]. However, more research is needed to reveal the relationship between alcohol consumption and DR.

There are several limitations to this study. First, the study was based on a cross-sectional survey that had several defects due to medical views. For example, BMI, physical activity status, FPG, and blood pressure could differ according to the time of measurement. Secondly, we did not distinguish between type 1 and type 2 diabetes mellitus. According to an epidemiologic study, in Korea, the incidence of type 1 diabetes mellitus is 0.6 per 100,000 which is very small number, while the incidence of type 2 is 8,290 per 100,000 [6,49]. Therefore, we assumed that all patients had type 2 diabetes mellitus. However, we cannot exclude the possibility that our findings were influenced by type 1 diabetic patients. Third, this is an Asian-specific study performed at the level of a single country. Generally, the incidence and progression of diabetes are influenced by ethnic differences and genetic backgrounds [43,50]. Thus, it is uncertain whether the results will be equally applicable to the general clinical practice.

## Conclusion

In summary, this study leads to the conclusion that sparse learning techniques using LASSO can contribute to the advancement of clinical decision-making tools with a good discriminative ability and to our understanding of risk factors for DR. This study supports that LASSO can be an effective prediction model not only in a bioinformatics problem, but also in the analysis of high-dimensional electronic health records. We hope that this study helps diabetic patients to reduce the risk of DR, which is the major cause of blindness in such patients.

## Competing interests

The authors declare that they have no competing interests.

## Authors’ contributions

EO collected the data, analyzed the experimental results, provided feedback on the paper, and revised the manuscript. TKY designed and conducted the experiments, analyzed the results, and drafted the research article. ECP collected the data, and provided feedback on the paper. All authors read and approved the final paper. EO and TKY contributed equally to this work.

## Pre-publication history

The pre-publication history for this paper can be accessed here:

http://www.biomedcentral.com/1472-6947/13/106/prepub

## Supplementary Material

Additional file 1 Regression coefficients of the classical logistic regression models and the sparse learning models trained with the training dataset. Click here for file

Additional file 2 Diagnostic performance of the LASSO models in the different scenarios, the support vector machine model, and the artificial neural network model. Click here for file
