# Supplementary material for: Diabetic retinopathy risk prediction for fundus examination using sparse learning: a cross-sectional study
Source: BMC Med Inform Decis Mak. 2013 Sep 13;13:106. doi: 10.1186/1472-6947-13-106 (PMC3847617; doi:10.1186/1472-6947-13-106)
Supplement: Additional file 1 — Regression coefficients of the classical logistic regression models and the sparse learning models trained with the training dataset. [file 1472-6947-13-106-S1.docx]

**Additional file 1 Regression coefficients of the classical logistic regression models and the sparse learning models trained with the training dataset.**

(A) Scenario 1

|  | OLR | LR-BS | Ridge | Elastic net | LASSO |
| --- | --- | --- | --- | --- | --- |
| Demographics |  |  |  |  |  |
| Sex (female) | 0.422 |  | 0.360 | 0.278 | 0.199 |
| Age (years) | -0.047 | -0.046 | -0.036 | -0.030 | -0.022 |
| Current smoke | 0.510 | 0.290 | 0.472 | 0.342 | 0.216 |
| Alcohol (>1 serving/week) | -0.230 |  | -0.206 | -0.125 | -0.039 |
| Physical activity (MET h/week) | 0.010 |  | 0.011 | 0.008 | 0.005 |
| Waist circumference (cm) | 0.008 |  | -0.001 |  |  |
| BMI (kg/m^2^) | -0.117 | -0.088 | -0.078 | -0.069 | -0.058 |
| Medical history |  |  |  |  |  |
| Duration of diabetes (years) | 0.062 | 0.057 | 0.059 | 0.056 | 0.054 |
| Diagnosed diabetes | 0.860 | 0.863 | 0.671 | 0.498 | 0.242 |
| Insulin therapy | 21.266 | 2.954 | 1.808 | 1.553 | 1.012 |
| Anti-diabetic drug | 20.201 | 1.850 | 0.801 | 0.541 |  |
| Nondrug anti-diabetic therapy | -18.374 |  | 0.807 | 0.917 |  |
| Diagnosed hypertension | -0.876 | -0.585 | -0.396 | -0.345 | -0.228 |
| Drug for hypertension | 0.302 |  | -0.071 |  |  |
| Diagnosed hyperlipidemia | 0.055 |  | 0.060 |  |  |
| Drug for hyperlipidemia | -0.318 |  | -0.271 | -0.130 | -0.036 |
| Blood pressure |  |  |  |  |  |
| Systolic BP (mmHg) | 0.019 | 0.020 | 0.014 | 0.007 |  |
| Diastolic BP (mmHg) | -0.031 | -0.035 | -0.024 | -0.016 | -0.007 |
| Intercept | -0.111 | 0.755 | -0.291 | -0.350 | -0.489 |

BMI, body mass index; BP, blood pressure; LASSO, least absolute shrinkage and selection operator; LR-BS, logistic regression with backward stepwise selection; OLR, ordinary logistic regression.

(B) Scenario 2

|  | OLR | LR-BS | Ridge | Elastic net | LASSO |
| --- | --- | --- | --- | --- | --- |
| Demographics |  |  |  |  |  |
| Sex (female) | 0.101 |  | 0.122 |  |  |
| Age (years) | -0.040 | -0.020 | -0.025 | -0.015 | -0.007 |
| Current smoke | 0.823 | 1.131 | 0.702 | 0.606 | 0.532 |
| Alcohol (>1 serving/week) | -0.564 | -0.661 | -0.404 | -0.274 | -0.159 |
| Physical activity (MET h/week) | 0.008 |  | 0.006 | 0.001 |  |
| Waist circumference (cm) | 0.007 |  | -0.002 |  |  |
| BMI (kg/m^2^) | -0.129 | -0.096 | -0.089 | -0.074 | -0.059 |
| Medical history |  |  |  |  |  |
| Duration of diabetes (years) | 0.029 |  | 0.031 | 0.028 | 0.027 |
| Diagnosed diabetes | 1.393 | 1.390 | 0.951 | 0.821 | 0.592 |
| Insulin therapy | 20.580 | 3.266 | 1.826 | 1.477 | 1.117 |
| Anti-diabetic drug | 19.312 | 1.772 | 0.663 | 0.314 |  |
| Nondrug anti-diabetic therapy | -17.566 |  | 0.905 | 1.050 |  |
| Diagnosed hypertension | -0.660 |  | -0.262 | -0.143 |  |
| Drug for hypertension | 0.283 |  | -0.034 |  |  |
| Diagnosed hyperlipidemia | -0.293 |  | -0.163 | -0.029 |  |
| Drug for hyperlipidemia | -0.072 |  | -0.155 | -0.148 |  |
| Blood pressure |  |  |  |  |  |
| Systolic BP (mmHg) | 0.022 |  | 0.014 | 0.005 |  |
| Diastolic BP (mmHg) | -0.038 |  | -0.027 | -0.015 | -0.007 |
| Blood test |  |  |  |  |  |
| FPG (mg/dL) | 0.012 | 0.017 | 0.010 | 0.010 | 0.009 |
| HbA1c (%) | 0.113 |  | 0.138 | 0.116 | 0.103 |
| Hemoglobin (g/dL) | -0.279 | -0.376 | -0.247 | -0.248 | -0.230 |
| Cholesterol (mg/dL) | 0.031 | 0.011 | 0.004 |  |  |
| TG (mg/dL) | -0.003 |  | 0.001 | 0.002 | 0.002 |
| HDL (mg/dL) | -0.037 |  | -0.013 | -0.006 | -0.003 |
| LDL (mg/dL) | -0.030 | -0.013 | -0.004 |  |  |
| AST (IU/L) | 0.002 |  | 0.003 |  |  |
| ALT (IU/L) | -0.001 |  | -0.002 |  |  |
| BUN (mg/dL) | 0.083 | 0.053 | 0.067 | 0.046 | 0.037 |
| Serum creatinine (mg/dL) | 1.059 |  | 0.650 | 0.092 |  |
| Intercept | 1.840 | 1.891 | 1.445 | 1.306 | 0.311 |

ALT, alanine aminotransferase; AST, aspartate aminotransferase; BMI, body mass index; BP, blood pressure; BUN, blood urea nitrogen; FPG, fasting plasma glucose; HbA1c, glycated hemoglobin; HDL, high-density lipoprotein; LASSO, least absolute shrinkage and selection operator; LDL, low-density lipoprotein; LR-BS, logistic regression with backward stepwise selection; OLR, ordinary logistic regression; TG, triglyceride.

(C) Scenario 3

|  | OLR | LR-BS | Ridge | Elastic net | LASSO |
| --- | --- | --- | --- | --- | --- |
| Demographics |  |  |  |  |  |
| Sex (female) | 0.132 |  | 0.149 |  |  |
| Age (years) | -0.060 | -0.047 | -0.034 | -0.024 | -0.012 |
| Current smoke | 0.831 | 0.774 | 0.663 | 0.555 | 0.469 |
| Alcohol (>1 serving/week) | -0.605 |  | -0.378 | -0.251 | -0.137 |
| Physical activity (MET h/week) | 0.004 |  | 0.005 | 0.002 |  |
| Waist circumference (cm) | 0.015 |  | -0.004 |  |  |
| BMI (kg/m^2^) | -0.207 | -0.159 | -0.112 | -0.099 | -0.082 |
| Medical history |  |  |  |  |  |
| Duration of diabetes (years) | 0.033 |  | 0.033 | 0.029 | 0.027 |
| Diagnosed diabetes | 1.149 | 1.353 | 0.822 | 0.664 | 0.427 |
| Insulin therapy | 20.711 | 3.125 | 1.708 | 1.377 | 0.956 |
| Anti-diabetic drug | 19.630 | 2.266 | 0.778 | 0.437 |  |
| Nondrug anti-diabetic therapy | -17.501 |  | 0.974 | 1.116 |  |
| Diagnosed hypertension | -1.613 | -0.491 | -0.372 | -0.101 |  |
| Drug for hypertension | 1.325 |  | 0.145 |  |  |
| Diagnosed hyperlipidemia | -0.219 |  | -0.085 |  |  |
| Drug for hyperlipidemia | -0.092 |  | -0.153 | -0.132 | -0.028 |
| Blood pressure |  |  |  |  |  |
| Systolic BP (mmHg) | 0.029 | 0.030 | 0.017 | 0.007 |  |
| Diastolic BP (mmHg) | -0.043 | -0.050 | -0.028 | -0.016 | -0.004 |
| Blood test |  |  |  |  |  |
| FPG (mg/dL) | 0.012 |  | 0.008 | 0.008 | 0.008 |
| HbA1c (%) | -0.012 | 0.402 | 0.063 | 0.051 | 0.054 |
| Hemoglobin (g/dL) | -0.303 | -0.320 | -0.247 | -0.263 | -0.256 |
| Cholesterol (mg/dL) | 0.017 |  | 0.003 |  |  |
| TG (mg/dL) | 0.000 |  | 0.002 | 0.002 | 0.002 |
| HDL (mg/dL) | -0.019 |  | -0.008 | -0.002 |  |
| LDL (mg/dL) | -0.020 |  | -0.005 | -0.001 |  |
| AST (IU/L) | -0.001 |  | -0.001 |  |  |
| ALT (IU/L) | 0.000 | 0.004 | 0.000 |  |  |
| BUN (mg/dL) | 0.090 |  | 0.066 | 0.047 | 0.037 |
| Serum creatinine (mg/dL) | 0.763 |  | 0.402 |  |  |
| Urine test |  |  |  |  |  |
| Protein (+) | 0.234 |  | 0.285 | 0.217 | 0.141 |
| Glucose (+) | 0.845 |  | 0.620 | 0.543 | 0.442 |
| Ketone (+) | -0.241 | -0.214 | -0.158 | -0.135 | -0.111 |
| Bilirubin (+) | 0.246 | 0.220 | 0.169 | 0.145 | 0.118 |
| Blood (+) | 0.440 | 0.346 | 0.352 | 0.234 | 0.096 |
| Urobilinogen (+) | -0.280 |  | 0.117 |  |  |
| Urine creatinine (mg/L) | -0.002 |  | -0.002 | -0.001 |  |
| Urine sodium (mmol/day) | -0.005 | -0.007 | -0.005 | -0.004 |  |
| Intercept | 5.613 | 3.156 | 3.689 | 3.295 | 2.047 |

ALT, alanine aminotransferase; AST, aspartate aminotransferase; BMI, body mass index; BP, blood pressure; BUN, blood urea nitrogen; FPG, fasting plasma glucose; HbA1c, glycated hemoglobin; HDL, high-density lipoprotein; LASSO, least absolute shrinkage and selection operator; LDL, low-density lipoprotein; LR-BS, logistic regression with backward stepwise selection; OLR, ordinary logistic regression; TG, triglyceride.
