# Supplementary material for: Diabetic retinopathy risk prediction for fundus examination using sparse learning: a cross-sectional study
Source: BMC Med Inform Decis Mak. 2013 Sep 13;13:106. doi: 10.1186/1472-6947-13-106 (PMC3847617; doi:10.1186/1472-6947-13-106)

**Additional file 2 Diagnostic performance of the LASSO models in the different scenarios, the support vector machine model, and the artificial neural network model.**

|  | AUC (95% CI) | Accuracy (%)  (95% CI) | Sensitivity (%)  (95% CI) | Specificity (%)  (95% CI) | PPV (%) | NPV (%) |
| --- | --- | --- | --- | --- | --- | --- |
| (A) Internal validation group (N = 163) | | | | | | |
| LASSO - Scenario 1 | 0.77 (0.70-0.83) | 70.0 (62.2-76.8) | 74.2 (66.6-80.6) | 68.9 (61.2-75.9) | 35.9 | 91.9 |
| LASSO - Scenario 2 | 0.80 (0.73-0.86) | 72.4 (64.8-79.0) | 77.4 (70.1-83.5) | 71.2 (63.5-77.9) | 38.7 | 93.1 |
| LASSO - Scenario 3* | 0.81 (0.74-0.86) | 73.6 (66.0-80.1) | 77.4 (70.1-83.5) | 72.7 (65.1-79.3) | 40.0 | 93.2 |
| SVM - Scenario 3^†^ | 0.83 (0.76-0.88) | 74.8 (67.3-81.2) | 71.0 (63.3-77.7) | 75.8 (68.3-82.0) | 40.7 | 91.7 |
| ANN - Scenario 3^‡^ | 0.79 (0.72-0.85) | 71.2 (63.5-77.9) | 80.6 (73.6-86.3) | 68.9 (61.2-75.9) | 37.9 | 93.8 |
| (B) External validation group (N = 562) | | | | | | |
| LASSO - Scenario 1 | 0.76 (0.73-0.80) | 71.7 (67.6-75.4) | 75.7 (71.8-79.2) | 70.6 (66.6-74.4) | 40.0 | 91.8 |
| LASSO - Scenario 2 | 0.80 (0.76-0.83) | 74.4 (70.5-78.0) | 73.0 (69.0-76.6) | 74.8 (70.9-78.4) | 42.9 | 91.5 |
| LASSO - Scenario 3* | 0.82 (0.78-0.85) | 75.2 (71.3-78.7) | 72.1 (68.0-75.8) | 76.0 (72.1-79.5) | 43.7 | 91.3 |
| SVM - Scenario 3^†^ | 0.81 (0.78-0.84) | 74.1 (70.1-77.7) | 75.7 (71.8-79.2) | 73.7 (69.7-77.3) | 42.6 | 92.1 |
| ANN - Scenario 3^‡^ | 0.79 (0.76-0.83) | 71.9 (67.8-75.6) | 81.1 (77.5-84.3) | 69.5 (65.4-73.3) | 40.7 | 93.4 |

* Data were shown in Table 4.

^†^ The SVM model was constructed by LIBSVM with all variables in scenario 3. The optimal model (Gaussian kernel function with a penalty parameter *C* of 10 and scaling factor *σ* of 50) was obtained by a grid search with 5-fold cross validation.

^‡^ The ANN model based on MATLAB 2010 was trained with all variables in scenario 3. The optimal model (5 nodes of a hidden layer and learning rate of 0.1) was obtained by a grid search with 5-fold cross validation.

ANN, artificial neural network; AUC, area under the receiver operating characteristic curve; CI, confidence interval; LASSO, least absolute shrinkage and selection operator; NPV, negative predictive value; PPV, positive predictive value; SVM, support vector machine.

(A) ROC curves in the internal validation group B) ROC curves in the external validation group


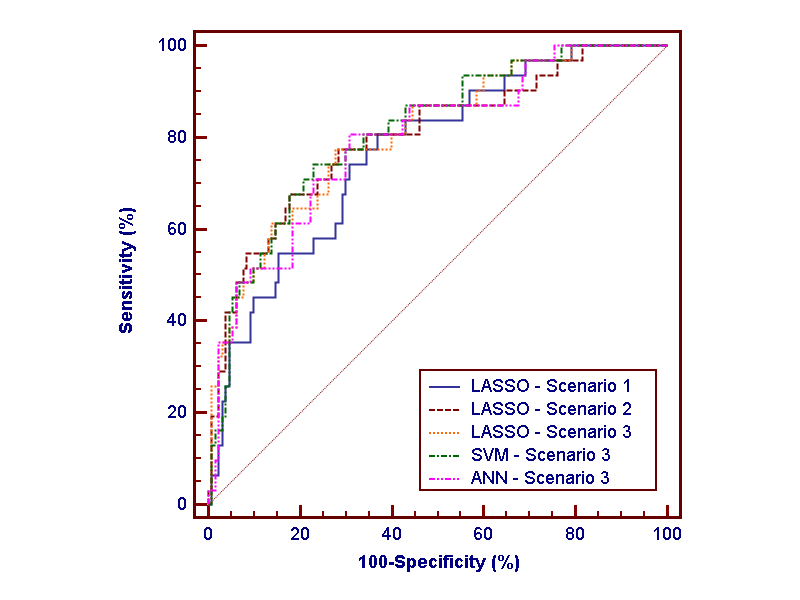

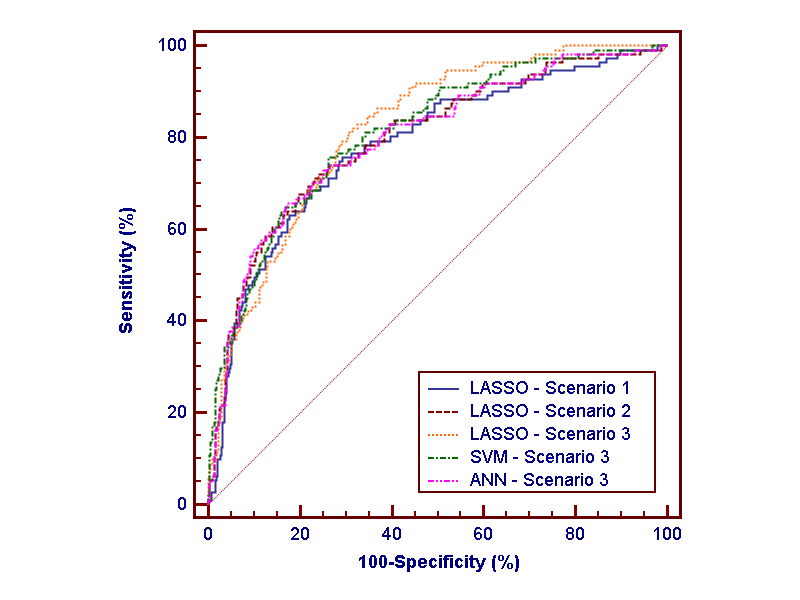

Supplement: Additional file 2 — Diagnostic performance of the LASSO models in the different scenarios, the support vector machine model, and the artificial neural network model. [file 1472-6947-13-106-S2.docx]
